# Sequence Depth, Not PCR Replication, Improves Ecological Inference from Next Generation DNA Sequencing

**DOI:** 10.1371/journal.pone.0090234

**Published:** 2014-02-28

**Authors:** Dylan P. Smith, Kabir G. Peay

**Affiliations:** Department of Biology, Stanford University, Stanford, California, United States of America; U.S. Geological Survey, United States of America

## Abstract

Recent advances in molecular approaches and DNA sequencing have greatly progressed the field of ecology and allowed for the study of complex communities in unprecedented detail. Next generation sequencing (NGS) can reveal powerful insights into the diversity, composition, and dynamics of cryptic organisms, but results may be sensitive to a number of technical factors, including molecular practices used to generate amplicons, sequencing technology, and data processing. Despite the popularity of some techniques over others, explicit tests of the relative benefits they convey in molecular ecology studies remain scarce. Here we tested the effects of PCR replication, sequencing depth, and sequencing platform on ecological inference drawn from environmental samples of soil fungi. We sequenced replicates of three soil samples taken from pine biomes in North America represented by pools of either one, two, four, eight, or sixteen PCR replicates with both 454 pyrosequencing and Illumina MiSeq. Increasing the number of pooled PCR replicates had no detectable effect on measures of α- and β-diversity. Pseudo-β-diversity – which we define as dissimilarity between re-sequenced replicates of the same sample – decreased markedly with increasing sampling depth. The total richness recovered with Illumina was significantly higher than with 454, but measures of α- and β-diversity between a larger set of fungal samples sequenced on both platforms were highly correlated. Our results suggest that molecular ecology studies will benefit more from investing in robust sequencing technologies than from replicating PCRs. This study also demonstrates the potential for continuous integration of older datasets with newer technology.

## Introduction

Next generation DNA sequencing (NGS) has changed the face of microbial ecology in the space of a few years. As a result, we have gained unprecedented insight into the community dynamics of morphologically cryptic organisms such as fungi, bacteria and viruses [1] [2] [3]. However, the outcome of NGS based ecological inquiry may be sensitive to technical practices that in many cases have not been adequately tested. For instance, the assumption that NGS read counts accurately reflect absolute abundance in ecological analyses may not be appropriate due to taxon specific PCR and sequencing biases [4]. These technical practices have important effects on our view of underlying biological reality, but also on the allocation of resources (time, money, reagents) that often define the scope of ecological inquiry.

Early optimization of NGS methods has focused on correcting platform specific sequencing issues, such as the known homopolymer error rates in 454 pyrosequencing [5], often with bioinformatic solutions. However, potential distortions may also arise prior to DNA sequencing during sample collection [6], DNA extraction [7] [8], or PCR amplification [9] [10]. Recognition of these problems has led to a loosely knit collection of best lab and bioinformatics practices that have emerged in the microbial ecology literature and that are aimed at increasing the robustness of whole community amplification. Among other things, such practices include the use of hot-start Taq polymerase, reducing the number of amplification cycles, and the pooling of multiple PCR replicates per sample [11]. The necessity of pooling PCR replicates is thought to arise from stochasticity in the PCR process that results in variable composition of DNA fragments across individual PCR reactions. Possible causes for this may be sampling effects that lead to variation in the initial population of DNA template used to start the reaction, slight variation in initial conditions, or priority effects of amplification in the early rounds of PCR. The few studies that have actually reported results from replicate NGS of the same sample (e.g. [8] [12]) have found sample-to-sample variance in sequence composition that seem to support the importance of stochastic PCR effects. However, these studies have focused on the comparison of individual PCR replicates that were sequenced separately, and therefore the extent to which their conclusions rely on PCR or sequencing stochasticity is still unknown.

Though many studies have suggested pooling PCR replicates prior to sequencing, to our knowledge no study has directly tested whether samples comprised of multiple, pooled PCR replicates capture a more robust sample of the true diversity within the sample. Because there have been no explicit tests of PCR pooling, papers vary wildly in the number of pooled PCR replicates they use or recommend. While three appears to be a somewhat canonical number (e.g. it has been adopted by the Earth Microbiome Project [13]) other studies have used a single PCR replicate [14], five [15] or even ten [16].

The rapid rise and fall of NGS sequencing platforms also raises major concerns about portability of data across studies. One of the major advantages of DNA based community profiling is the collection of standardized data that can be compared or combined across studies. However, if the observed structure of a community is platform dependent it would seriously weaken the additive nature of sequencing efforts in microbial ecology [17].

In this study our primary goals were (1) to determine quantitatively the number of pooled PCR replicates that maximizes ecological inference in NGS studies and (2) to test the robustness of ecological inferences about community structure using two different NGS sequencing platforms. We did this using a two-pronged experimental approach where the same DNA sample was sequenced from a pool of 1, 2, 4, 8 or 16 separate PCR reactions using both Roche's 454 Pyrosequencing (454) and Illumina's MiSeq (MiSeq). We then compared patterns of α- and β-diversity (the primary response variables in most community ecology studies) among samples and replicates. In addition, we used both NGS platforms to sequence a larger set of soil samples taken from Pine forests in geographically distinct parts of North America where we expected to see differences in community composition.

Based on previous studies, we hypothesized that increasing the number of PCR replicates prior to sequencing would increase α-diversity, reduce β-diversity and increase reproducibility by averaging out PCR noise. Surprisingly, we found that increasing PCR replication did not meaningfully change any of our ecological response variables and may be a poor investment of resources in molecular ecology studies. By contrast, increased sequencing depth markedly improved estimates of β-diversity using both NGS platforms. In addition, we found that community sequencing results from 454 and MiSeq provide largely similar results, suggesting that data from the two platforms can be combined in a meaningful way.

## Materials and Methods

### Experimental design

We investigated the effects of PCR replication and sequencing platforms on ecological inference using samples from an ongoing project to characterize ectomycorrhizal fungal communities across North America pine forests. To test the hypothesis that pooling PCR replicates improves ecological inference, we selected three soil samples taken from two sites in Oregon (42.79° E –121.62° N) and one in Connecticut (41.82° E, –72.96° N). To test whether NGS sequencing platform affects ecological inference, we selected 60 samples from two sites in North Carolina (NC1 36.01° E, –78.97° N, NC2 35.99° E, –79.10° N), two sites in California (CA1 37.84° E, –119.94° N, CA2 37.81° E, –119.91° N), and two sites in Alaska (AK1 64.77° E, –148.27° N, AK2 64.76° E, –148.25° N) (for all sites n = 10).

All sample sites in Oregon and California were located on United States Department of Agriculture (USDA) National Forest land. The Alaskan sites were located at the Bonanza Creek Long Term Ecological Research (LTER) site. Sites in North Carolina were located on private land owned by Duke University, and sites in Connecticut were located in the Gold's Pine State Forest. No permits were required for any site, and all necessary permissions were obtained prior to sampling. Soil samples were taken from either the homogenized organic or mineral layer of a core approximately 7.5 cm diameter ×14 cm deep (**File S1**). Soils were stored cool until ∼0.25 g were extracted using the Powersoil DNA extraction kit (MoBio, Carlsbad CA). DNA extracts were diluted 1:20 and then 1 µl used for PCR.

### Molecular methods

For sequencing using the 454 platform, PCR was carried out using modified versions of the fungal specific primer set ITS1F [18] and ITS4 [19]. The 5′ end of the ITS1F primer was modified to include the 454 Lib-L A adapter plus a 10-bp molecular identification (MID) tag to allow for sample multiplexing as in [14]. The 5′ end of the ITS4 primer was modified to include the 454 Lib-L B adapter.

For sequencing using the Illumina MiSeq platform, we designed modified versions of the primer set ITS1F and ITS2 [19]. This primer set targets a shorter section of the fungal ITS region because of the shorter read lengths possible with MiSeq. The 5′ end of the ITS1F primer was modified to include the forward Illumina Nextera adapter and a two basepair “linker” sequence designed to mismatch against all major fungal lineages immediately upstream of the gene primer (**Fig S1**). The induced mismatch is designed to decrease potential taxon-specific PCR bias from downstream matches to the adapter or barcode. The 5′ end of the ITS2 primer was modified with the appropriate reverse Illumina Nextera adapter, linker sequence, and a 12-bp error-correcting Golay barcode as in [17]. Using the program NetPrimer (Premier Biosoft, Palo Alto CA) we designed three custom sequencing primers that demonstrated low dimerization potential and high thermodynamic compatibility with each other and with the Illumina-specific PhiX sequencing primer. The Read 1 and Read 2 sequencing primers were designed to anneal to the gene priming regions of the amplicons and extend further into the conserved 18S portion of the amplified region, thereby maximizing the amount of ITS sequence returned by the reads. The Index sequencing primer was designed to sequence only the 12 bp barcode of each amplicon.

PCR was carried out in 25 µl reactions including 1 µl genomic DNA, 0.5 µl of each 10 µM primer, 5 µl of 5× OneTaq Standard Reaction Buffer (New England BioLabs, Ipswitch MA), 0.5 µl of 10 mM dNTPs (New England BioLabs, Ipswitch MA), and 0.63 units Taq polymerase. All PCR reactions were set up on ice and using Fusion hot start Taq polymerase (New England Biolabs, Ipswitch MA) to minimize non-specific amplification and primer dimerization. PCR conditions were: denaturation at 94°C for 1 min; 30 amplification cycles of 30 sec at 94°C, 30 sec at 52°C and 30 sec at 68°C; followed by a 7 min final extension at 68°C. PCR products were visualized using gel electrophoresis and successful samples cleaned using the Agencourt Ampure XP kit (Beckman Coulter, Brea CA). For the replication experiment, the three samples were each amplified 1, 2, 4, 8 or 16 times using a separate MID tag or barcode for each replication treatment (N = 3 samples ×5 replication levels  =  15). Individual PCR reactions for a given sample × replication treatment were pooled and then 20 µl of each pool cleaned using the Ampure Kit as above.

Cleaned PCR products were quantified using the Qubit hs-DS-DNA kit (Invitrogen, Carlsbad CA) on a Tecan Infinite F200 Pro plate reader reading at 485 nm excitation and 530 nm emission. PCR products to be sequenced with 454 were then pooled in equimolar concentration and sent to the Duke University Institute for Genome Sciences & Policy core and sequenced on a ¼ plate partition using Titanium FLX chemistry. PCR products generated for Illumina sequencing were pooled at equimolar concentration and then multiplexed with 44 additional bacterial samples containing 16S rDNA amplicons used for an unrelated study. The final pool containing both loci was sent to the Stanford Functional Genomics Facility for 250 bp paired-end sequencing on an Illumina MiSeq. Bacterial and fungal sequencing primers were also pooled for each read before submission to the sequencing facility. A spike of 30% PhiX was included in the amplicon library in order to achieve sufficient sample heterogeneity. Raw sequence data are deposited at NCBI's Short Read Archive under study accession SRP035367. Sample metadata information is provided as File S1.

### Bioinformatics

Sequence de-multiplexing and bioinformatic processing of the 454 and Illumina datasets were performed using aspects of the QIIME [20] and the UPARSE [21] pipelines. Initial quality filtering of 454 sequences excluded all sequences <350 or >1200 bp, with any primer mismatches, with a homopolymer run >10 bp, or with a mean quality score below 25. The remaining sequences were denoised using flowgram clustering [22]. Pre-filtered forward and reverse reads from the Illumina dataset were 238 bp long and our multiplexing strategy resulted in high quality sequences for both fungal and bacterial samples. For the fungal samples analyzed in this study, reads were trimmed with CutAdapt [23] to the point where the sequence met the distal priming site, and further trimmed using Trimmomatic [24] to remove any additional low quality end regions. After quality trimming forward reads averaged 208 bp and reverse reads averaged 185 bp. Reads were paired using USEARCH v. 7.0.1001 with a minimum Phred score sequence cutoff threshold of 3 and a minimum sequence length of 75 bp. Paired reads averaged 230 bp and were discarded if they contained >0.25 expected errors. The final fasta file containing all sequences used for analysis is available from the authors upon request.

All final, high-quality sequences from both the 454 and Illumina datasets were combined and grouped into operational taxonomic units (OTUs) in USEARCH using the UPARSE-OTU and UPARSE-OTUref algorithms (which included chimaera detection and filtering and dropped all global singleton reads) at a 97% sequence similarity cutoff. OTUs were given taxonomic assignments in QIIME based on a previously published sequence database [8] modified for QIIME compatibility as in [25]. To compare samples on an equal basis all samples were rarefied to even sampling depths prior to statistical analysis. Rarefaction depths were determined ad-hoc to maximize the number of samples included while still maintaining a reasonable number of sequences. For the replication experiment, the 454 samples were rarefied to 500 sequences and the Illumina samples to 38,000 sequences. For the larger, cross platform-comparison dataset, 454 samples were rarefied to 1000 sequences and the Illumina samples to 40,000 sequences.

### Statistical analysis

To see how replication affects ecological inference we calculated a number of common α-diversity (observed richness, Fisher's Alpha, Chao 1 and Simpson, Simpson Evenness) and β-diversity (Jaccard, Bray-Curtis, β-sim) metrics used in community ecology. We used a linear model to test whether or not the number of PCR replicates and sequencing platform affected different richness estimators (S  =  Replication × Sample ID × Platform). We used a similar approach to test whether average β-diversity changed in any predictable way with the number of replicates used to generate each sample. This was done by calculating β-diversity (Bray Curtis or Jaccard) for each replication level compared with all other samples sequenced from those plots.

This dataset also allowed us a unique look at data reproducibility with repeated sequencing of the same sample. To see how sequencing depth affects estimates of sample β-diversity, we calculated within sample β-diversity (that is, β-diversity between independent replicates of the same sample – hereafter termed pseudo-β-diversity) at a range of sequencing depths, from 50–1000 (454) and 100 to 80,000 (Illumina). Because there has been much debate about the handling and validity of low abundance OTUs, we tested the effect of within sample sequence abundance on the repeatability with which an OTU is detected across replicate sequencing of the same sample. We used logistic regression to model the relationship between log_10_ transformed mean within sample abundance and frequency of detection across samples (for this analysis 0 values were assigned ½ the minimum observed value prior to log transformation). We also looked at quantitative reproducibility by comparing OTU read abundance (log_10_ X+1 transformed) between the single replication treatment and the 16 replication treatment for each DNA sample.

Finally, with the larger dataset we compared the similarity of ecological inferences made with different sequencing platforms. α-diversity estimates were generated for each sample based on rarefaction to a common sequence depth within each platform. We used a Mantel test to determine whether community similarity estimates were similar across platforms. Pairwise sample similarity across samples and across platforms was visualized using non-metric dimensional scaling (NMDS), and a perMANOVA tested for the effect of sequencing platform and geographic origin on estimates of community similarity. To compare whole community overlap we generated a Venn diagram to illustrate the proportion of shared and unique taxa generated with each platform. To look for taxonomic bias we plotted relative abundance of lineages for shared and unique OTUs across platforms. Statistics were performed using the R software package [26] and the Vegan community analysis package for NMDS and perMANOVA [27].

Several data points were left out of our analyses due to either insufficient or low-quality sequences or sample mishandling (454 data: the 2 PCRs treatment for OR1, the 8 PCRs treatment for OR4, and individual points CA1.A5.OH and CA1.A5.AH from the larger dataset. MiSeq data: the 4 PCRs treatment for OR1 and individual point NC2.0.OH from the larger dataset).

## Results

After quality control, denoising, and chimera removal of the smaller dataset, sequencing depth for successful samples ranged from 573–1783 sequences in the 454 dataset and from 38,423–92,189 sequences in the Illumina dataset. Observed richness at 500 sequences (454 dataset) ranged from approximately 30 to 60 OTUs/sample and at 38,000 sequences (Illumina dataset) ranged from approximately 200 to 350 OTUs/samples. As expected for Pine soils the most commonly observed taxa belonged to lineages of Basidiomycota ectomycorrhizal fungi and saprotrophs (data not shown).

**Figure 1:**
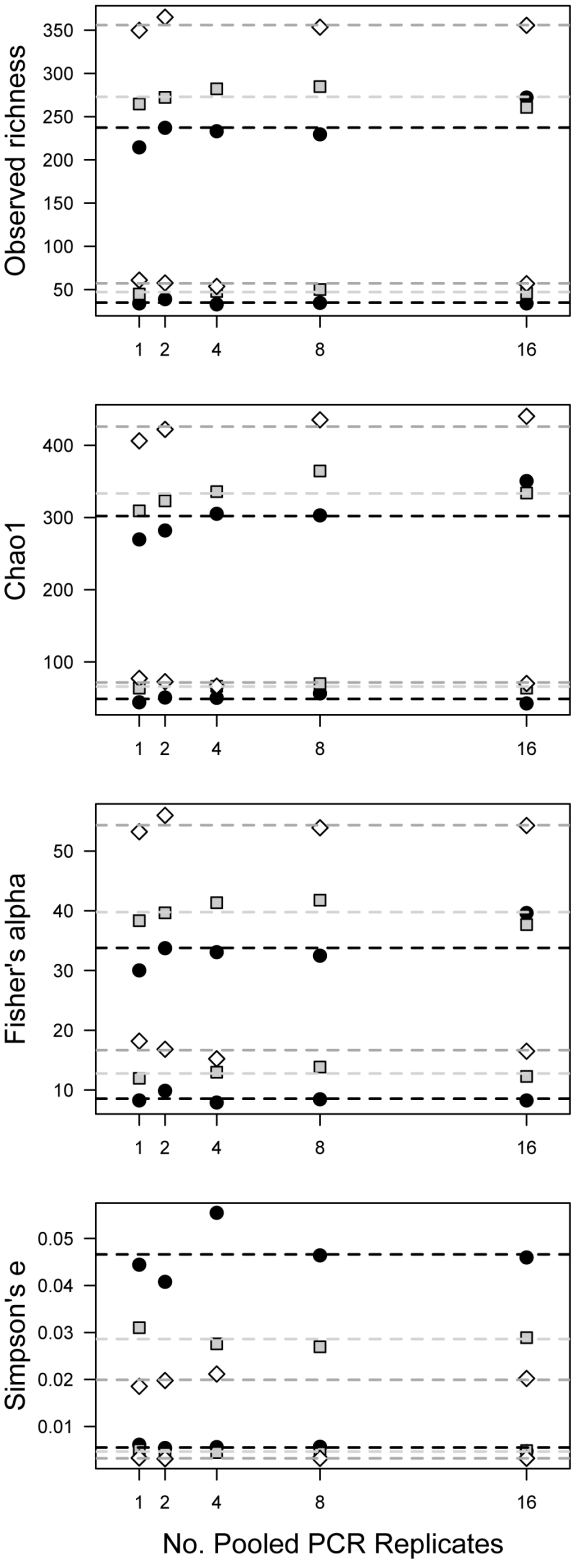
Estimated species richness does not depend on the number of PCR reactions pooled prior to sequencing. Plots of independent replicates representing different levels of PCR pooling for samples CT2, OR1, and OR4 against four different diversity indicators. Points are colored by sample ID. Dotted lines represent the average between different replicates of the same sample. The top three lines represent samples sequenced with Illumina MiSeq and the bottoms three lines represent the same samples sequenced with 454.

Increasing the number of PCR replicates pooled prior to sequencing had no effect on the estimated α-diversity of a sample regardless of the sequencing method used (**Table 1**). This effect was consistent regardless of the richness metric chosen (**Fig 1**) and whether or not an interaction term was included in the model. Similarly, estimates of β-diversity compared with other samples in the same site did not show any trends related to the number of pooled PCR replicates (454 Jaccard ANOVA F_1,7_ = 0.588, P = 0.468; Bray-Curtis ANOVA F_1,7_ = 1.682, P = 0.236; Illumina Jaccard ANOVA F_1,8_ = 0.118, P = 0.741; Bray-Curtis ANOVA F_1,8_ = 1.685, P = 0.231; **Fig S2**). That is to say, a sample sequenced from 1 PCR did not show higher or lower β -diversity with other samples from the same plot than the same sample sequenced from 16 PCR replicates. When ordinated the different replication levels from the same sample clustered together with little variation from the centroid (**Fig S2**).

**Table 1:** Analysis of variance tests for an effect of PCR replicate number, sample identity, and sequencing method on fungal richness from the CT2, OR1, and OR4 samples.

	No. PCR Replicates		Sample ID		Method		Replicates × Sample ID		Replicates × Method	
	F_1,22_	*P*	F_2,22_	*P*	F_1,22_	*P*	F_2,22_	*P*	F_1,22_	*P*
**Observed**	0.372	0.548	18.748	<0.001*	646.450	<0.001*	0.320	0.730	0.261	0.615
**Chao1**	2.380	0.137	15.480	<0.001*	717.970	<0.001*	0.171	0.844	2.428	0.134
**Fisher's Alpha**	0.415	0.526	38.256	<0.001*	490.635	<0.001*	0.439	0.650	0.430	0.519
**Simpson**	0.060	0.809	42.190	<0.001*	17.250	<0.001*	0.185	0.832	0.000	0.991
**Simpson's E**	0.001	0.977	13.502	<0.001*	137.701	<0.001*	0.054	0.947	0.001	0.979

Samples were sequenced with both 454 and Illumina MiSeq.

**Figure 2:**
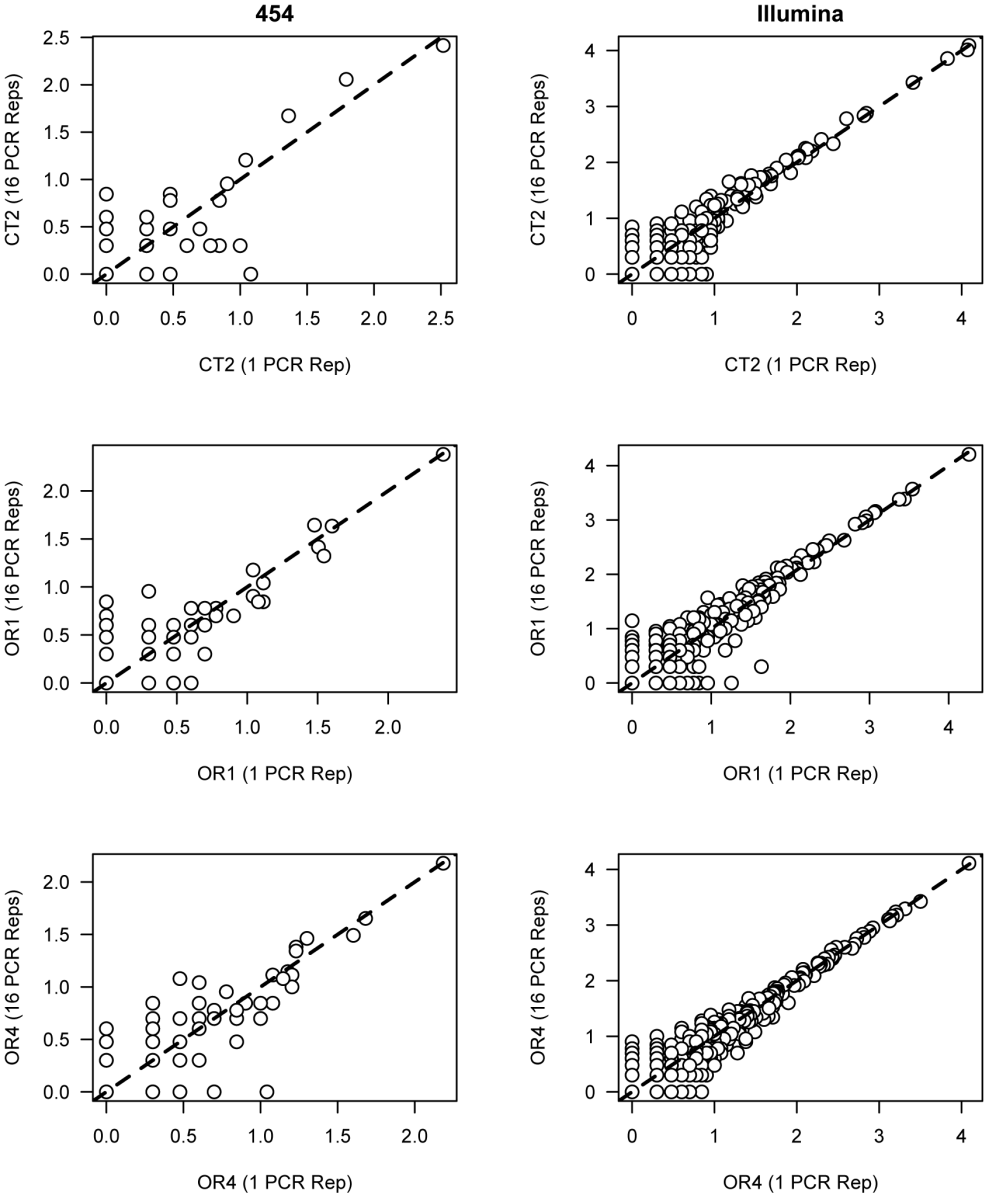
Taxon abundance is strongly correlated across sequencing runs with different levels of PCR replication. Circles represent individual taxa, and the relationship between the log_10_ of their abundance in samples comprised of 16 PCR replicates (y axis) and one PCR replicate (x axis) for CT2 (top two panels), OR1 (middle two panels), and OR4 (bottom two panels). Dashed lines represent a 1∶1 relationship. The left three panels show samples sequenced with 454 and the right three panels show samples sequenced with Illumina MiSeq.

Sequence counts for individual OTUs were highly correlated across resequencing instances of the same sample. This relationship was true regardless of the PCR replicate number. Plots relating the number of sequences per taxon in a sample composed of 16 PCR replicates to that of a single PCR replicate were strongly log linear and in almost all cases followed a 1∶1 relationship (**Fig 2**). As a result, per OTU sequence counts were highly significantly correlated (Pearson's product moment correlation: 454 dataset CT2 r = 0.84, P<0.001; OR1 r = 0.89, P<0.001; OR4 r = 0.87, P<0.001; Illumina dataset CT2 r = 0.92, P<0.001; OR1 r = 0.94, P<0.001; OR4 r = 0.96, P<0.001).

**Figure 3:**
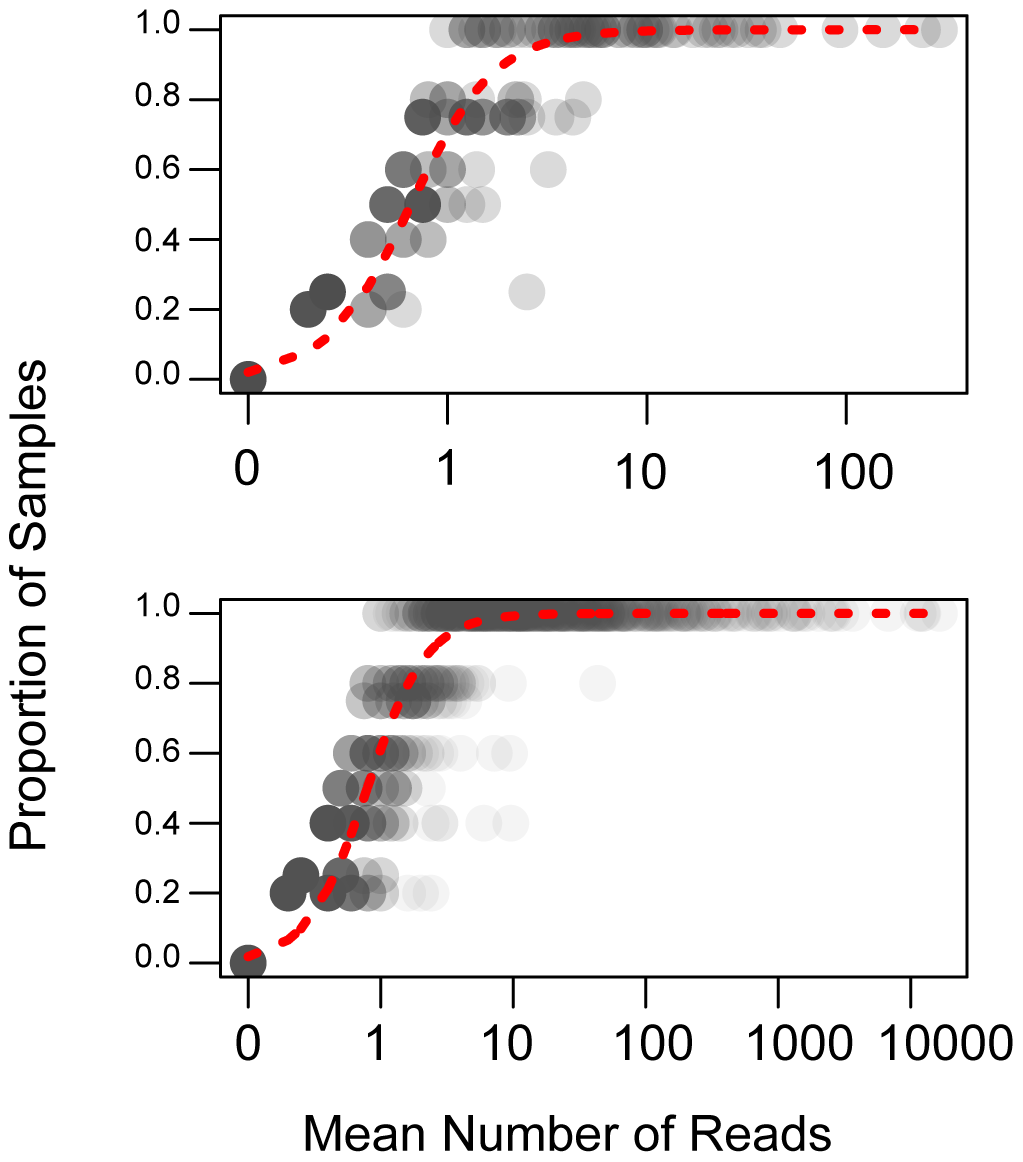
Taxa represented by more sequences are observed most consistently across replicates of the same sample. Plots of the proportion of samples in which an OTU is observed against the average number of reads representing that OTU. Zeros were assigned a value of 0.1 prior to log transformation. Dashed lines represent the logistic model predicting the relationship. The top panel shows results from 454 and the bottom panel shows results from Illumina.

High abundance OTUs were detected more consistently across replicate sequencing runs. The proportion of samples that an OTU was observed in increased significantly with average within sample read depth for that taxon (**Fig 3**; Overall effects tests: 454 No. reads χ^2^_1_ = 1,496, P<0.001, Site χ^2^_2_ = 1.0, P = 0.60; Illumina No. reads χ^2^_1_ = 10,742, P<0.001, Site χ^2^_2_ = 2.3, P = 0.32). There were no differences in this relationship across samples and the same patterns were seen for analyses run with median, maximum and minimum read depth (data not shown). For both 454 and Illumina OTUs with mean read abundance >10 sequences were detected nearly 100% of the time. However, many low abundance OTUs were also detected with a high degree of regularity.

**Figure 4:**
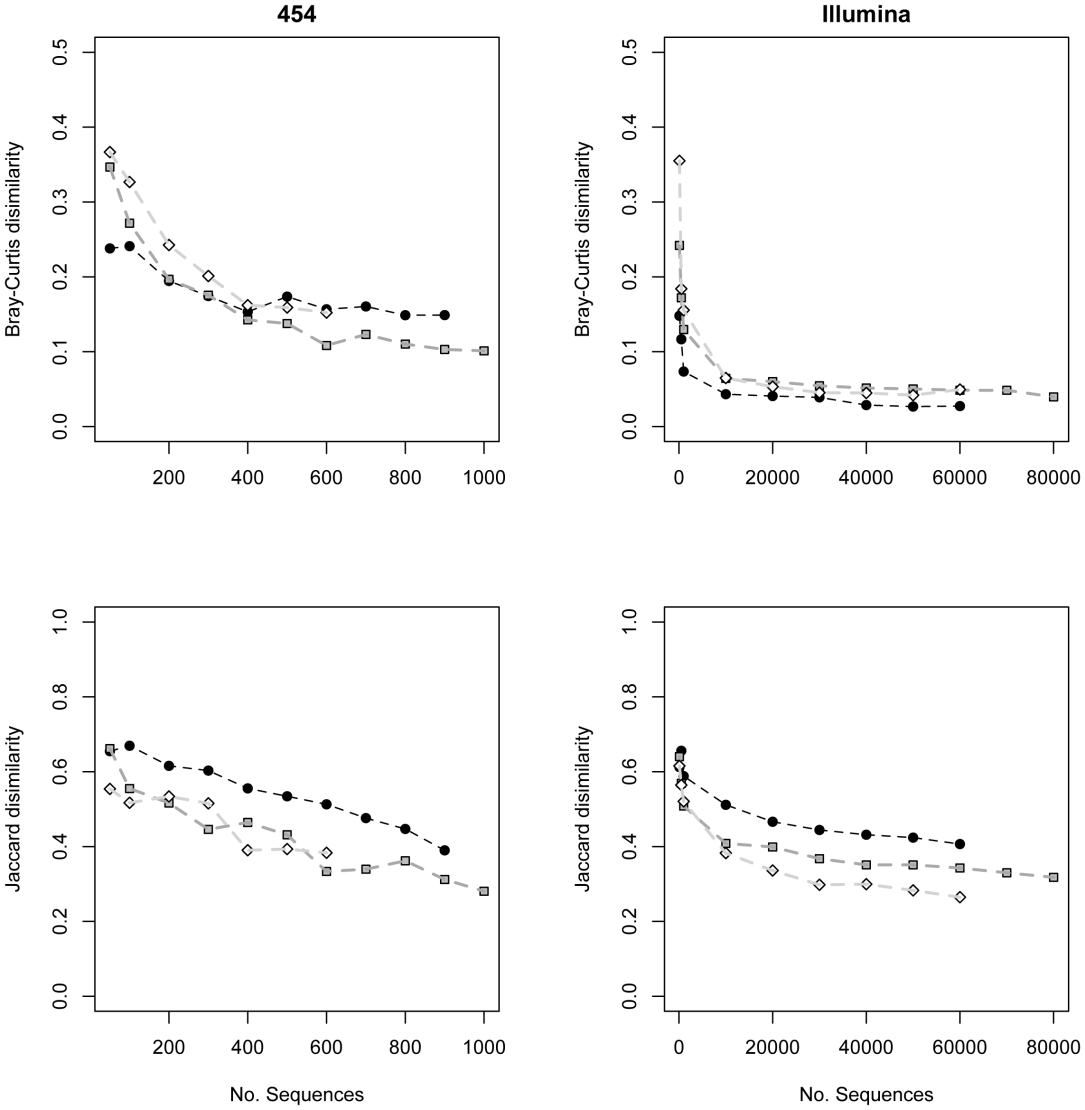
Increasing sequence depth reduces pseudo-β-diversity. Plots of the between-sample Bray-Curtis dissimilarity (top two panels) and Jaccard dissimilarity (bottom two panels) in CT2, OR1, and OR4 against per-sample sequencing depth. Points represent the β-diversity values between different replicates of the same sample and are colored by sample ID. Dashed lines connect each symbol within a sample. The left two panels show samples sequenced with 454 and the right two panels show samples sequenced with Illumina MiSeq.

Pseudo-β-diversity estimates decreased exponentially with increasing sequencing depth (**Fig 4**). In the 454 dataset, dissimilarity measures consistently remained above 0.1 (Bray-Curtis) and 0.3 (Jaccard) at the maximum amount of sequences/sample recovered. In the Illumina dataset, Bray-Curtis dissimilarity approached zero above 20,000 sequences/sample while Jaccard dissimilarity remained above 0.3 at the maximum amount of sequences/sample recovered.

**Figure 5:**
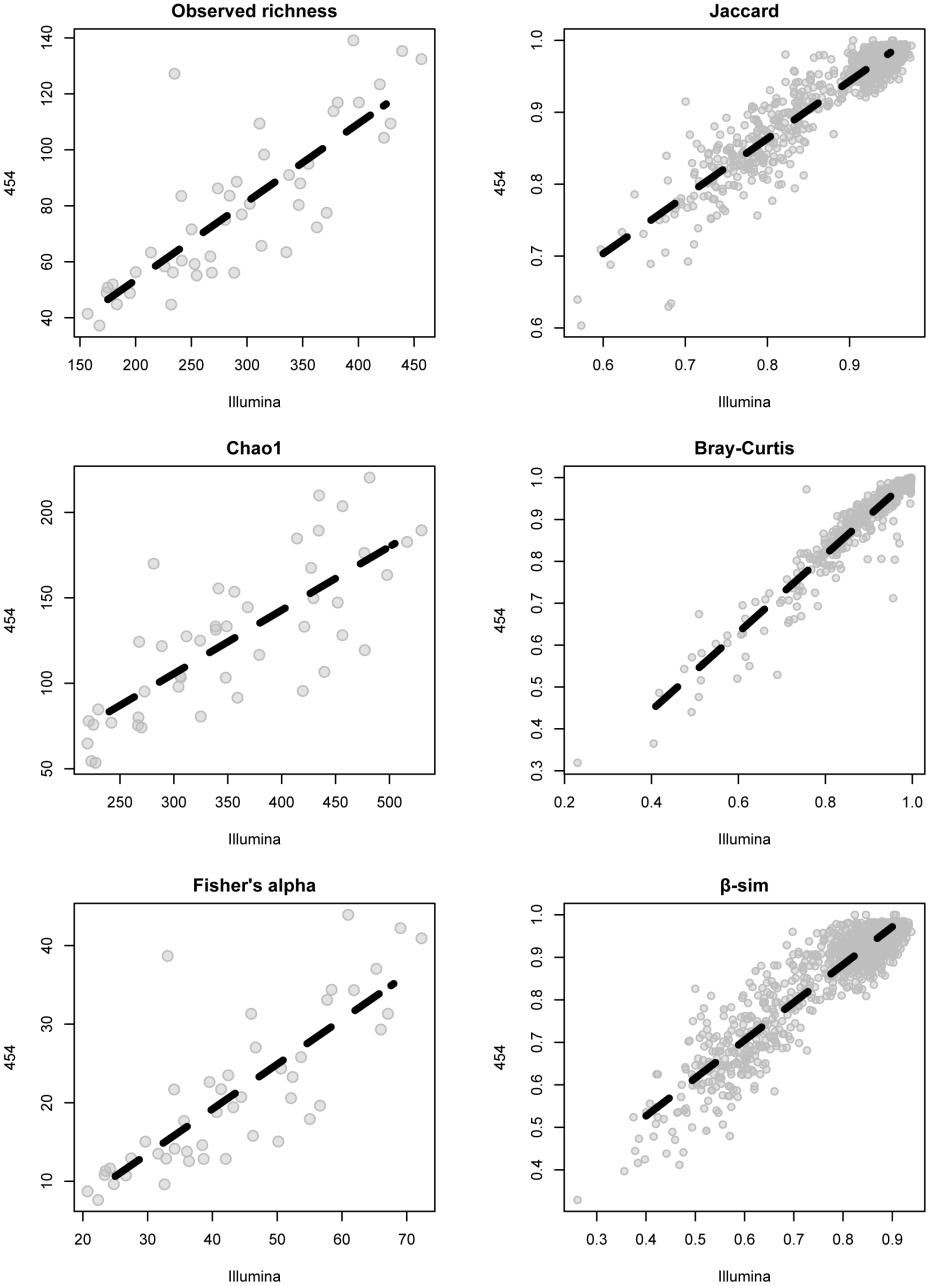
Patterns of α- and β-diversity are highly reproducible when samples are sequenced on different platforms. Regressions of diversity found in 55 soil samples sequenced on both platforms. Left three columns: points represent individual samples, and the relationship between the total richness per sample found when sequenced with 454 (y axis) and Illumina MiSeq (x axis). Right three columns: points represent pairwise differences in between-sample community composition and the relationship between dissimilarity found with 454 (y axis) and Illumina (x axis). Dashed lines represent the linear models predicting the relationships.

From the larger set of samples, sequencing on the 454 and Illumina MiSeq platforms resulted in 3,660 total OTUs. Of these 3 (0.08%) were unique to the 454 dataset, 1,798 (49.13%) were unique to the Illumina dataset, and 1,859 (50.79%) were shared (**Fig S3**). Richness was significantly higher for samples sequenced with Illumina MiSeq than for the same samples sequenced with 454 (**Table 2**). Regressions of per-sample observed species, Chao1 estimated richness, and Fisher's Alpha index between the 454 dataset and the Illumina dataset indicated that diversity recovered with either sequencing method was highly correlated (**Fig 5**: Observed slope = 0.28±0.03, r^2^ = 0.67, P<0.001; Chao1 slope = 0.37±0.05, r^2^ = 0.59, P<0.001, Fisher's Alpha slope  = 0.57±0.06, r^2^ = 0.65, P<0.001).

**Table 2:** Average per-sample fungal richness for 55 soil samples from pine forests.

	454 Avg. Richness/Sample	Illumina Avg. Richness/Sample	F_1,102_	*P*
**Observed**	79.907	289.000	322.800	<0.001*
**Chao1**	125.835	349.610	269.600	<0.001*
**Fisher's Alpha**	21.190	42.522	82.110	<0.001*
**Simpson**	0.814	0.819	0.032	0.858
**Simpson's E**	0.019	0.005	83.970	<0.001*

Samples were sequenced with both 454 and Illumina MiSeq.

**Figure 6:**
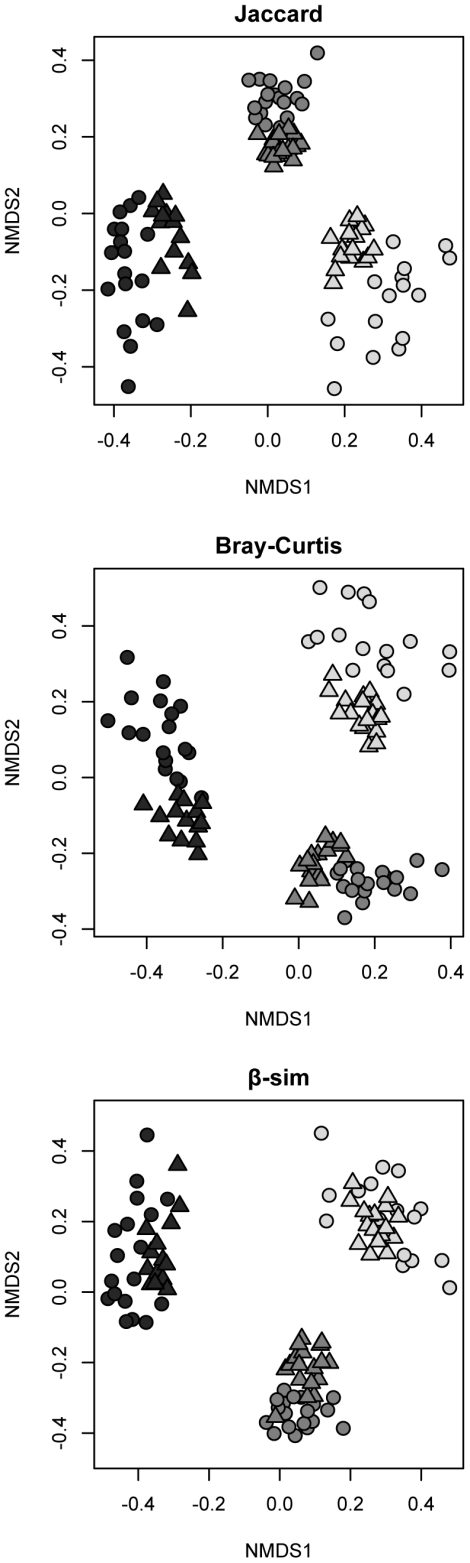
Broad ecological patterns of β-diversity are recovered equally well with each sequencing platform. Non-metric multidimensional scaling of fungal communities from 55 soil samples sequenced with both 454 (circles) and Illumina MiSeq (triangles). Points are colored by the three regions of sample collection. Ordinations are based on between-sample dissimilarity calculated with Jaccard (top panel), Bray-Curtis (middle panel), and β-sim (bottom panel).

β-diversity estimates of between-sample dissimilarity were somewhat affected by sequencing platform (Jaccard perMANOVA F_1,98_ = 30.26, r^2^ = 0.08 P = 0.001; Bray-Curtis perMANOVA F_1,94_ = 45.40, r^2^ = 0.09, P = 0.001; β-sim perMANOVA F_1,94_ = 29.30, r^2^ = 0.05, P = 0.001). However, regressions of β-diversity between the 454 dataset and the Illumina dataset showed that between-sample dissimilarity was highly correlated between the two sequencing platforms (**Fig 5**: Jaccard slope = 0.80±0.01, Mantel r = 0.94, P = 0.001; Bray-Curtis slope = 0.93±0.01, Mantel r = 0.96, P = 0.001, β-sim slope = 0.92±0.01, Mantel r = 0.92, P = 0.001). Both sequencing methods recovered the significant differences in community structure expected between sampling bioregions (Jaccard perMANOVA F_2,98_ = 124.40, r^2^ = 0.62, P = 0.001; Bray-Curtis perMANOVA F_2,98_ = 145.32, r^2^ = 0.61, P = 0.001; β-sim perMANOVA F_2,98_ = 204.45, r^2^ = 0.74, P = 0.001) which explained an order of magnitude more variation than did sequencing method. All samples from both sequencing datasets ordinated primarily by sampling bioregion (**Fig 6**).

**Figure 7:**
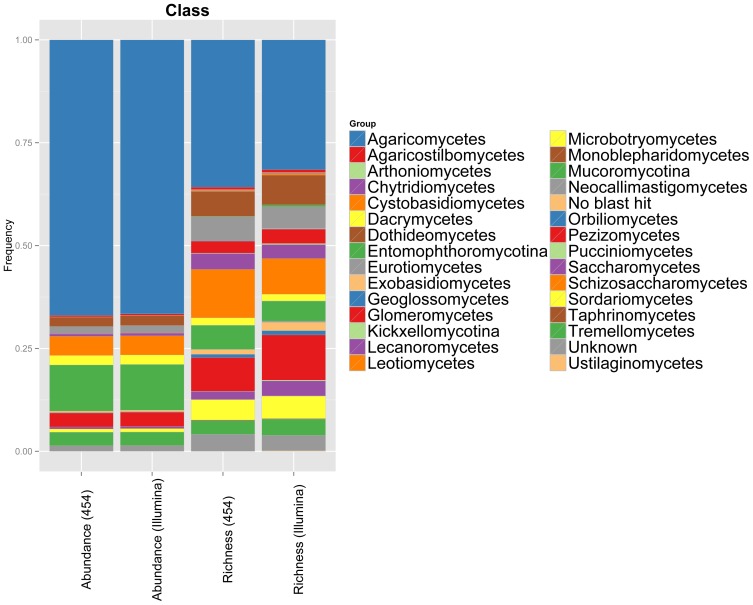
Taxonomic assignment to OTUs observed between both sequencing platforms is highly consistent. Bar chart indicating the proportional richness and abundance of taxa identified to the class level in 55 soil samples sequenced with both 454 and Illumina MiSeq.

Taxonomic assignment was highly consistent between OTUs found in the 454 dataset and OTUs found in the Illumina dataset at the phylum, class, and ordinal levels (**Fig 7** **, Fig S4**). Taxonomic bias between the two sequencing platforms was primarily limited to low abundance taxa (e.g. relatively more species of Zygomycota and Chytridomycota taxa found in the 454 dataset), but taxonomic composition of each dataset looked nearly identical when relative abundance of taxonomic groups was considered.

## Discussion

In this study we compare the effects of different lab, sequencing and bioinformatic protocols on a number of ecological metrics of α- and β-diversity. These metrics form the basis for conclusions in most microbial community studies and so our results should have important ramifications for how this work is carried out. This is particularly true because of the limited resources and options that can be explored in a single study.

Despite the popularity of PCR replication in molecular ecology studies, we find that increasing the number of PCR replicates that are pooled prior to sequencing has no meaningful effect on ecological measures of diversity or community structure and thus likely no effect on the conclusions of a given study. We present several lines of evidence to support this conclusion. First, the effect of PCR replication is highly insignificant in all statistical models predicting α- and β-diversity of replicates of three soil samples taken from different pine biomes across North America (**Table 1**, **Fig 1**, **Fig S2**). Although visually there appears to be a slight increase in species richness, Chao1 richness, and Fisher's Alpha diversity as more PCR replicates are pooled, the trend does not hold in a linear fashion and is both sample, sequencing method, and metric-specific (e.g. only seen in the Illumina dataset, only true for OR1 and OR4, and is most apparent only for Chao1 richness estimates). On the other hand, sample ID (i.e. sampling location, a more relevant ecological factor) has a comparably large effect on α- and β-diversity metrics. In all models tested, sample ID and sequencing method are the predominant drivers between differences in diversity and community structure.

Second, the number of sequences observed for each taxon between low-replicated samples (e.g. 1 PCR replicate) and high-replicated samples (16 PCR replicates) is highly correlative in a 1∶1 relationship (**Fig 2**), suggesting that pooling more PCR replicates prior to sequencing does not affect the relative abundances of taxa found in each sample, and that sequence abundance per taxon in one PCR can accurately predict sequence abundance per taxon in a pool of 16 replicates. It is important to note that the relationship between sequence abundance per taxon in high vs. low replicated samples is weakest with low-abundance taxa, highlighting the importance of adequate sequencing depth for obtaining an accurate depiction of diversity within samples. Together, these results suggest that ecological studies focused on comparing diversity levels and differences between multiple samples across gradients or treatments varying in space or environmental conditions will not be improved significantly by using multiple PCR replicates.

In searching through the literature the origins of this practice are actually somewhat unclear. In general, most evidence in favor of PCR replication is based on differences in OTUs detected in repeated sequencing of single PCR replicates of the same sample [8] [11] [12]. However, we would argue that sampling error during PCR is a small problem compared with sampling error in the actual sequencing process, in which a few thousand molecules are down sampled from an overall population of billions. Our data show that replicates of the same sample sequenced multiple times at low sequencing depth can lead to higher levels of between-replicate dissimilarity – i.e. pseudo-β-diversity - than should ideally be the case. This is most likely due to inadequate sequencing depth or suboptimal levels of rarefaction making low-abundance taxa unlikely to be appear in all samples. We find that the proportion of replicates of the same sample in which an OTU is present increases logarithmically with the average number of reads representing that OTU (**Fig 3**). This is to say that the reproducibility of taxon coverage and composition of a sample will improve with increasing sequencing depth per taxon. For both 454 and Illumina, OTUs represented by an average of >10 sequences are detected nearly 100% of the time. While many low abundance OTUs are detected repeatedly across samples and thus likely to be real, restricting analyses to these core OTUs may restrict the influence of pseudo-β-diversity due to limited sampling when making ecological conclusions about similarity of microbial communities.

In addition, we find that pseudo-β-diversity between sequencing replicates of the same sample decreases with an increasing number of sequences per sample (**Fig 4**). Pseudo-β-diversity of abundance sensitive metrics like Bray-Curtis dissimilarity decreases exponentially as more sequences are added, approaching zero (indicating little to no difference between replicates of the same sample) at a depth of >10,000 sequences/sample. In our study, sequencing on the 454 platform was unable to capture this sequencing depth and thus dissimilarity between replicates in this dataset remain higher (>0.1) at the maximum sequencing depth recovered. Sequencing on the Illumina platform recovered approximately 70× more sequences per sample and thus easily reaches the lowest Bray-Curtis dissimilarity values possible between replicates within the recovered sequencing depth. Interestingly, values of a binary metric like Jaccard dissimilarity remain higher than might be desired for multiple replicates of the same sample even at the maximum sequencing depth recovered for both platforms. This indicates that rare taxa continue to be detected in low abundance as more sequences are recovered on either platform, regardless of the total amount of additional sequences.

The implications of this result are several. First, it could suggest that extremely low-abundance microbial taxa are always present in high diversity systems such as soils. As technology progresses to achieve orders of magnitude more sequences per sample with each new sequencing platform, microbial ecology studies will tend to detect more and more rare taxa, perhaps without ever saturating taxa-accumulation curves. Using binary metrics like Jaccard could lead to artificially high estimates of between sample dissimilarity given how low-abundance and rare these new taxa are, and thus should be used with caution. Perhaps more realistically, the rare taxa that persist in detection at extremely low abundance as sequencing depth grows could also be a result of sequencing error and spurious OTU formation during sequence processing [21] [28] [29] [30]. As a result, the ability to saturate taxa-accumulation curves by increasing sequencing depth could be somewhat confounded by subsequent increases in sequencing or processing errors. However, given that we observed many low-abundance taxa in different replicates of the same sample (**Fig 3**), it is likely that many of these taxa represent real organisms. Again, we would argue that this should encourage microbial ecologists to consider relative sequence abundance when examining β-diversity comparisons between highly diverse samples. While there is no silver bullet, the right choice of metrics will depend on the relative risk of pseudo-β-diversity vs. taxon bias in addressing the particular ecological question at hand.

Our results from the larger dataset of soil samples from three different North American pine biomes reveal interesting insights about the influence of sequencing platform on ecological conclusions and thus the adaptability of microbial ecology studies to the latest NGS platforms like Illumina MiSeq. Since MiSeq reads are at present maximum only 300 bp in either direction, adapting ecological sequencing studies to newer technology presents challenges for longer loci previously sequenced with 454. Often this means designing new primers or switching loci altogether, and the degree to which similar patterns and conclusions can be drawn from sequencing the same organisms as labs adapt their protocols for the future remains uncertain. In our study, the 454 dataset and Illumina dataset differ in the reverse PCR primers used and in the length of the amplicons.

Sequencing of the same samples on the Illumina platform vs. the 454 platform results in a ∼40× increase in high quality read coverage as well as considerably more total OTUs and levels of richness per sample (**Table 2**). Encouragingly, the Illumina dataset finds nearly all OTUs that are detected with 454 but largely expands the total taxonomic coverage (**Fig S3**). The only three OTUs unique to the 454 dataset are identified as taxa in the genera Trichophaea (Ascomycota), Camarophyllopsis (Basidiomycota), and Amanita (Basidiomycota). Interestingly, eight Trichophaea taxa and 14 Amanita taxa are also present in the Illumina dataset, indicating no explicit lineage bias of the Illumina primers against members of these genera. The unique Thrichophaea and Amanita OTUs found only in the 454 dataset are thus likely due to sampling effects or error in the OTU clustering step of the sequencing processing pipeline. Additionally, since the read pairing step of Illumina processing is designed to correct sequencing errors from one read with higher-quality base calls from the other [31], it is possible that the 454-uniqe OTUs simply represent sequencing error that was not otherwise corrected.

Despite the large increase in diversity recovered with Illumina vs. 454, values of α- and β-diversity remain highly correlative between the two sequencing platforms (**Fig 5**). Sample bioregion is the predominant driving factor in the ordination of all samples from both sequencing methods (**Fig 6**), with 454 and Illumina replicates of the same samples clustering strongly by their sampling location. Within each bioregion cluster, samples cluster further by sequencing method. Variation around the centroid is greater for the 454 dataset, suggesting that β-diversity relationships between samples will depend slightly on the method used to sequence samples. This is likely due to differences in sequencing depths and species richness attainable with the two platforms. Some of the platform-specific β-diversity differences disappear in the ordination based on the β-sim metric, which controls for differences in richness between samples and thus a major difference with varied sequencing depth. It is important to note, however, that a certain degree of variation in ordination seen between the two datasets is to be expected given the different primer sets used for each platform. Still, our results strongly suggest that larger scale patterns of α- and β-diversity are as equally and consistently recoverable with newer, Illumina sequencing technology as with older 454 methods, and that ecologists should be able to transition their research with little hesitation to newer, more high-resolution sequencing technology.

Taxonomic assignment to OTUs is also consistent across the two sequencing platforms (**Fig 7** **, Fig S4**). At the phylum level, we observe almost complete agreement in the number and types of taxa identified. Differences between taxonomic assignment in the two datasets are primarily in the number of different Neocallimastigomycota, Glomeromycota, and Chytridiomycota taxa present. However, these groups represent a proportionally small amount of the total sequences recovered, and thus differences in the abundance of each taxonomic group recovered by the two datasets are accordingly quite small and could be due to stochasticity rather than bias. This relationship additionally holds true for the class and order groups.

Despite it's advent several years ago, amplicon sequencing with Illumina for higher order eukaryotic organisms like fungi remains scarce in the literature (but see [12] and [32]). Our results from the larger pine biome dataset present novel evidence that large scale ecological patterns of diversity, structure, and taxonomic resolution are easily attainable with an Illumina-specific fungal ITS primer set, and that ecological studies have much to gain by adopting newer NGS methods.

## Supporting Information

Figure S1 **Primer constructs for the amplification and sequencing of ITS1 for Illumina MiSeq.** a) Sequences of PCR and sequencing primers designed to amplify and sequence ITS1, specific to the Illumina MiSeq platform. b) Partial diagram of the ITS region in fungi (not to scale), with approximate annealing locations of PCR and sequencing primers. The PCR primers are designed to generate large amplicons comprising the variable ITS1 region and conserved 18S and 5.8S regions. The Read 1 and Read 2 sequencing primers are designed to sequence a smaller region comprised mostly of ITS1, eliminating most of the conserved flanking regions. The Index sequencing primer sequences the barcode on each amplicon.
(TIF)

Figure S2 **Pseudo-β-diversity is not significantly affected by the number of PCR replicates pooled prior to sequencing.** The top four panels show the average between-replicate dissimilarity between independent replicates of CT2, OR1, and OR4 plotted against increasing PCR replication level, as determined by sequencing with 454 and Illumina MiSeq. The bottom two panels show non-metric dimensional scaling (NMDS) ordinations of the same dissimilarity values. Different colored symbols represent the different sample IDs; different shaped symbols represent the PCR replication level of each replicate.
(TIF)

Figure S3 **Sequencing on the Illumina platform greatly expands the detectable taxonomic diversity.** Venn diagram of the total fungal OTUs found from 55 soil samples sequenced with both Illumina MiSeq (light grey circle) and 454 (dark grey circle). OTUs found only when samples were sequenced with Illumina or 454 are represented by the non-overlapping regions of the circles on the left (1798 OTUs) and right (3 OTUs), respectively. OTUs present in both sequencing runs are represented by the overlapping region in the middle (1859 OTUs).
(TIF)

Figure S4 **Taxonomic assignment to OTUs observed between both sequencing platforms is highly consistent.** Bar charts indicating the proportional richness and abundance of taxa in 55 soil samples sequenced with both 454 and Illumina MiSeq at the phylum, class, and order levels.
(TIF)

File S1 **Metadata for all samples collected and sequenced for this study.**
(TXT)
