# Supplementary figures and images for: Sequence Depth, Not PCR Replication, Improves Ecological Inference from Next Generation DNA Sequencing
Source: PLoS One. 2014 Feb 28;9(2):e90234. doi: 10.1371/journal.pone.0090234 (PMC3938664; doi:10.1371/journal.pone.0090234)

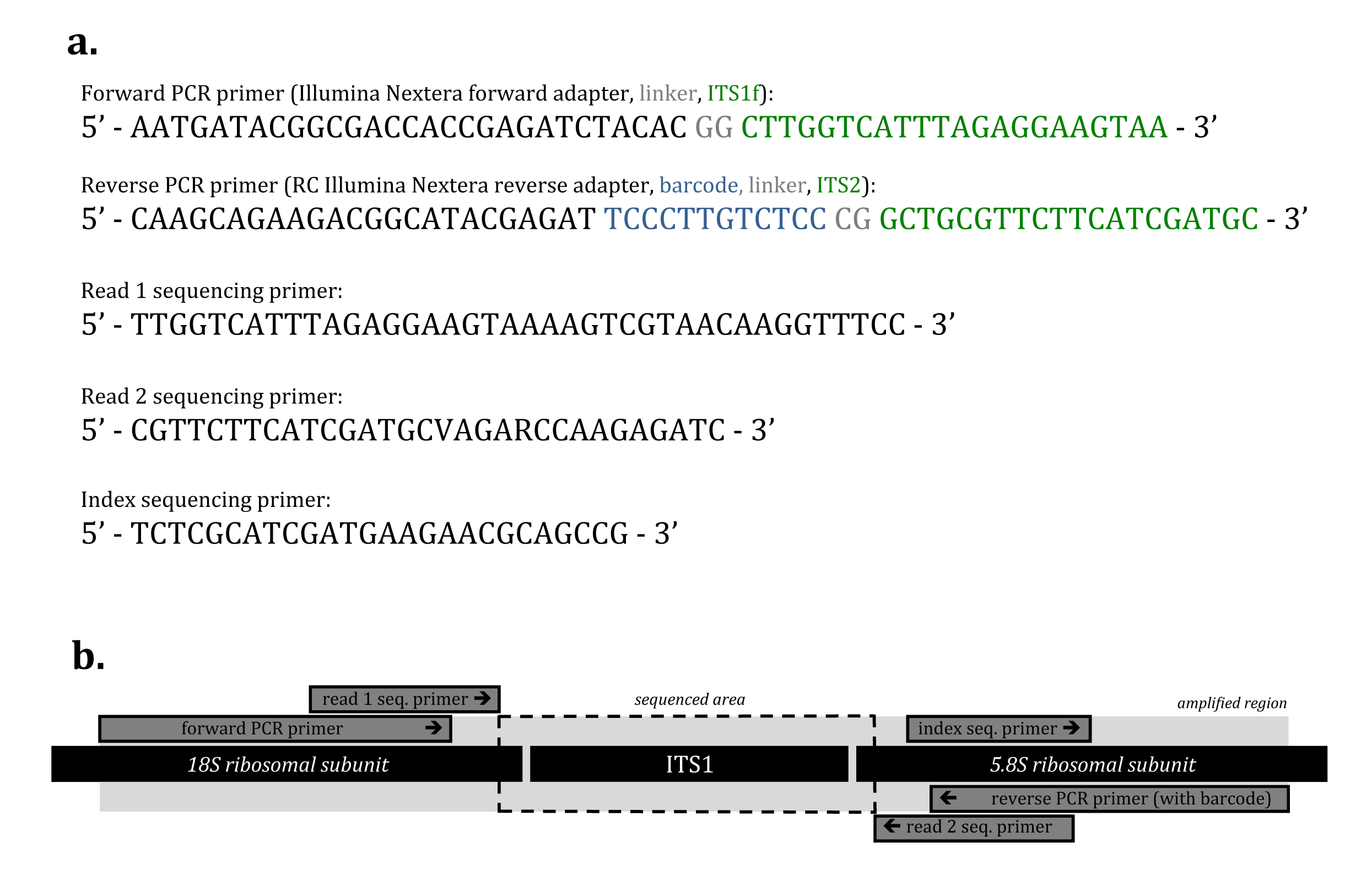

Supplement: Figure S1 — Primer constructs for the amplification and sequencing of ITS1 for Illumina MiSeq. a) Sequences of PCR and sequencing primers designed to amplify and sequence ITS1, specific to the Illumina MiSeq platform. b) Partial diagram of the ITS region in fungi (not to scale), with approximate annealing locations of PCR and sequencing primers. The PCR primers are designed to generate large amplicons comprising the variable ITS1 region and conserved 18S and 5.8S regions. The Read 1 and Read 2 sequencing primers are designed to sequence a smaller region comprised mostly of ITS1, eliminating most of the conserved flanking regions. The Index sequencing primer sequences the barcode on each amplicon. (TIF) [file pone.0090234.s001.tif]

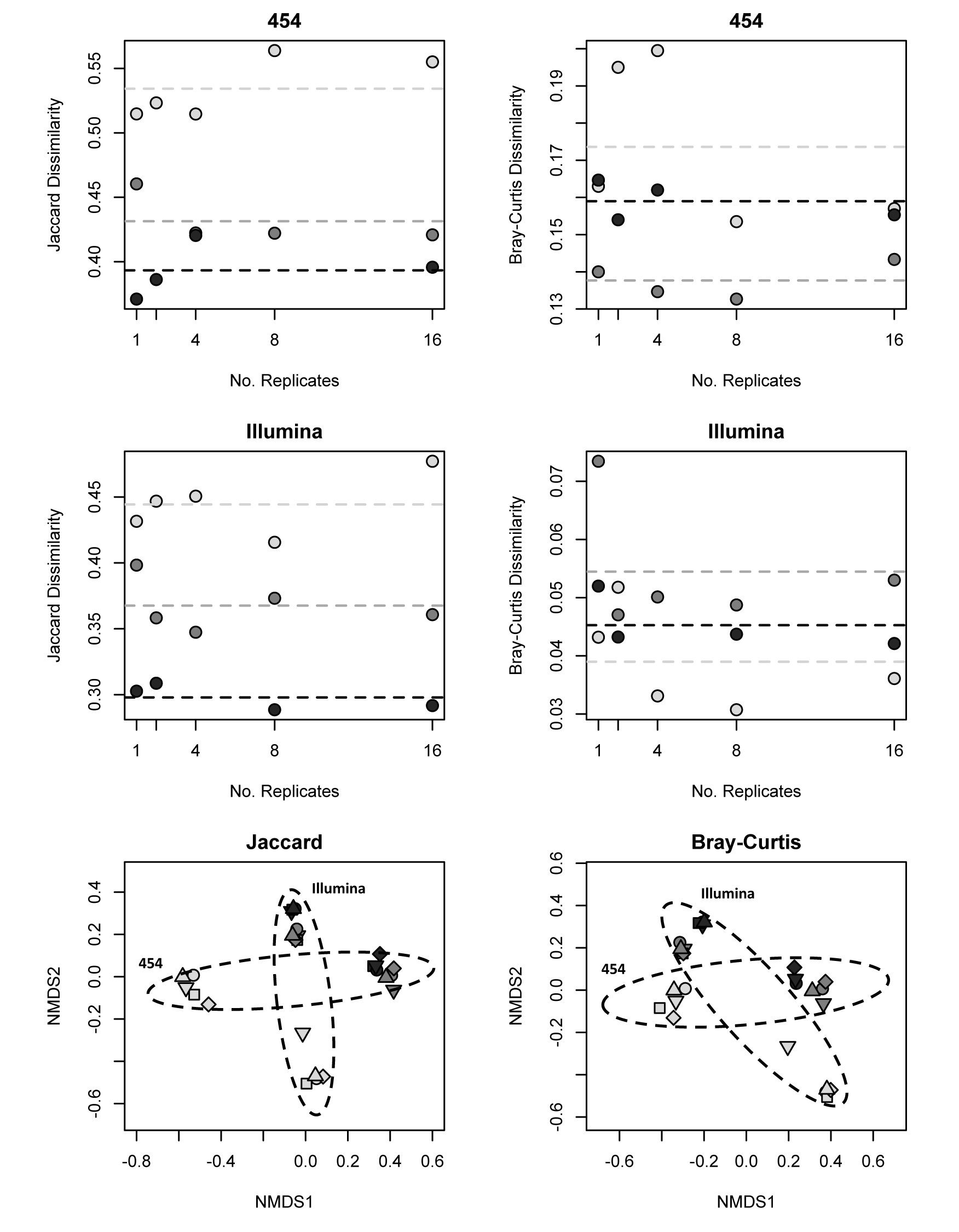

Supplement: Figure S2 — Pseudo-β-diversity is not significantly affected by the number of PCR replicates pooled prior to sequencing. The top four panels show the average between-replicate dissimilarity between independent replicates of CT2, OR1, and OR4 plotted against increasing PCR replication level, as determined by sequencing with 454 and Illumina MiSeq. The bottom two panels show non-metric dimensional scaling (NMDS) ordinations of the same dissimilarity values. Different colored symbols represent the different sample IDs; different shaped symbols represent the PCR replication level of each replicate. (TIF) [file pone.0090234.s002.tif]

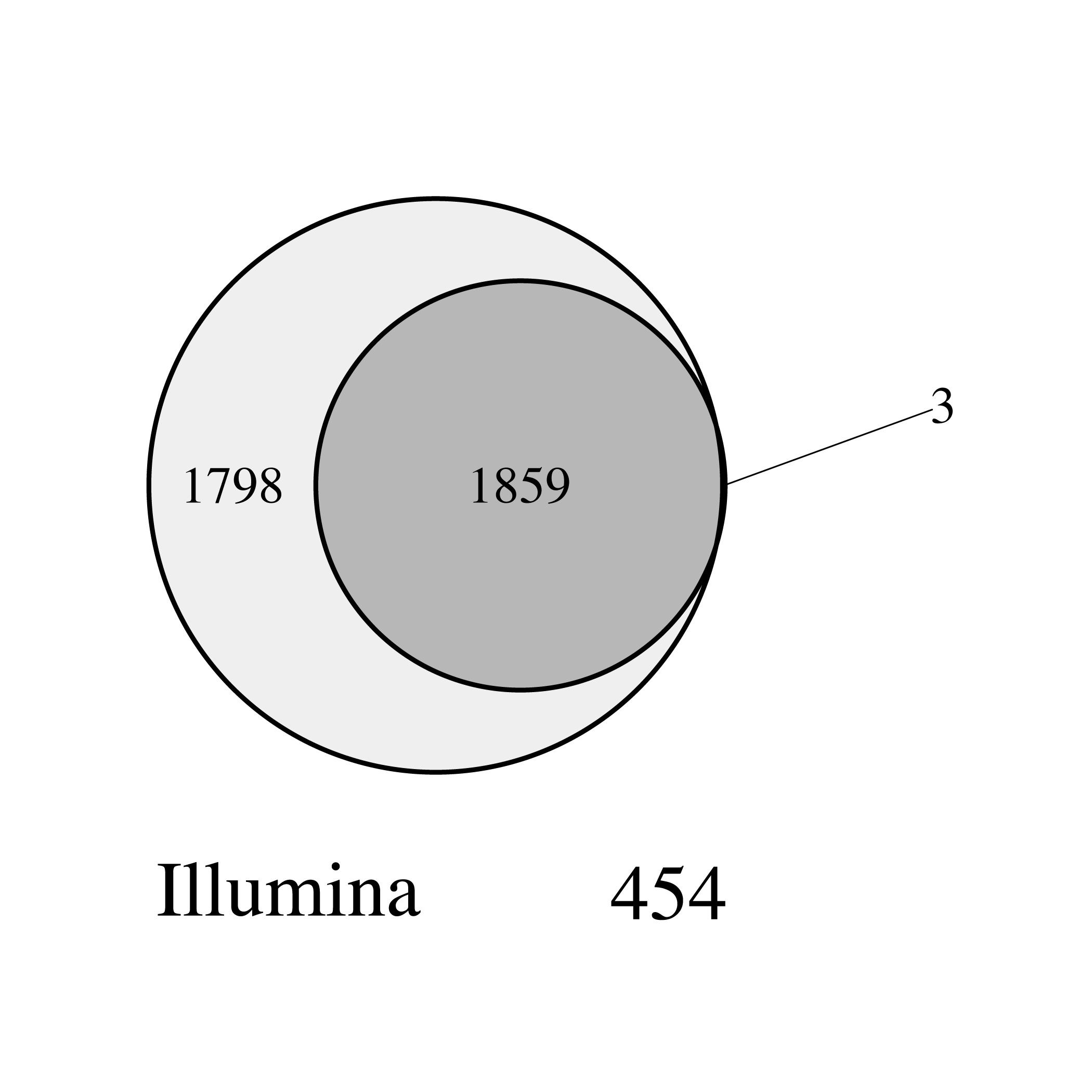

Supplement: Figure S3 — Sequencing on the Illumina platform greatly expands the detectable taxonomic diversity. Venn diagram of the total fungal OTUs found from 55 soil samples sequenced with both Illumina MiSeq (light grey circle) and 454 (dark grey circle). OTUs found only when samples were sequenced with Illumina or 454 are represented by the non-overlapping regions of the circles on the left (1798 OTUs) and right (3 OTUs), respectively. OTUs present in both sequencing runs are represented by the overlapping region in the middle (1859 OTUs). (TIF) [file pone.0090234.s003.tif]

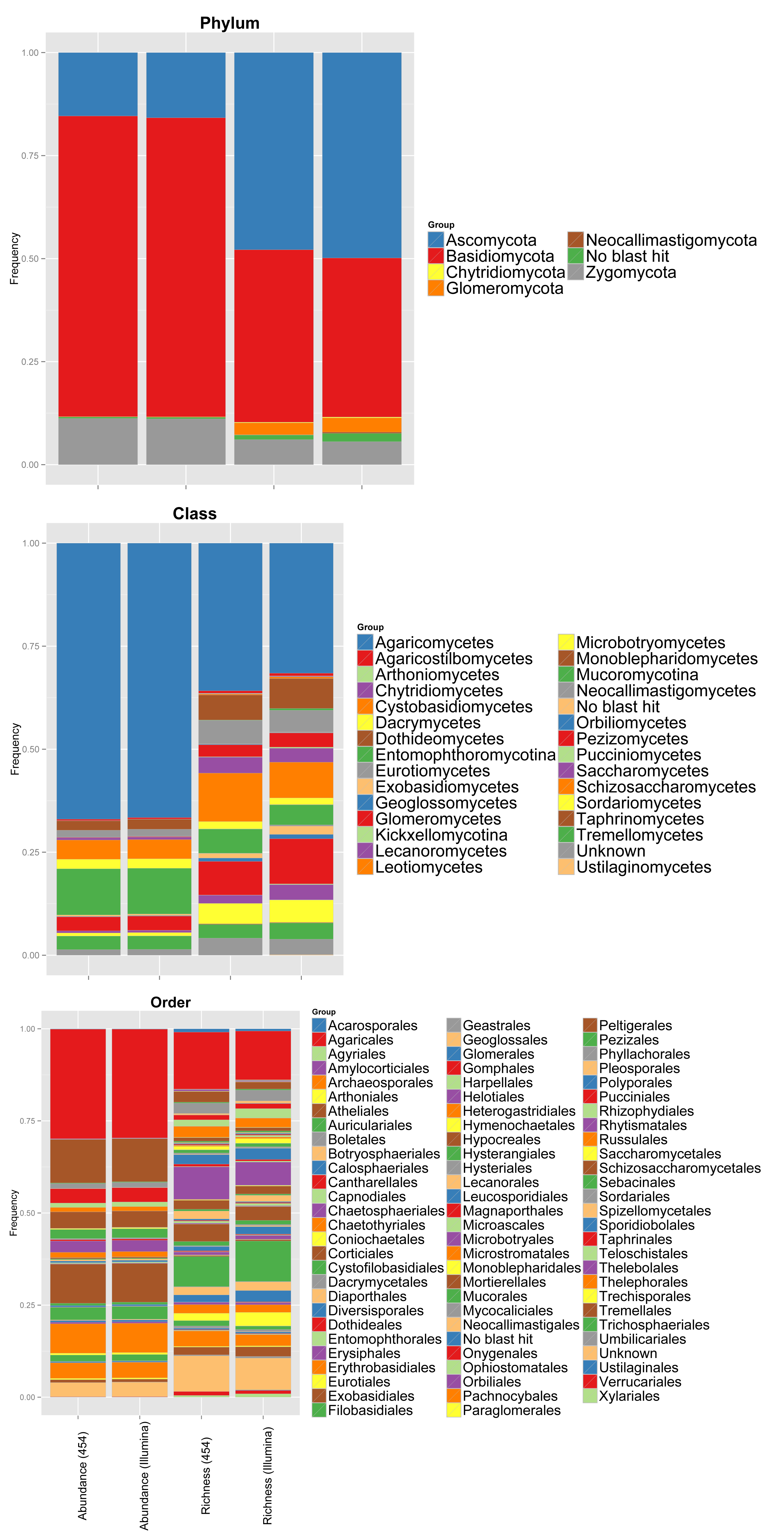

Supplement: Figure S4 — Taxonomic assignment to OTUs observed between both sequencing platforms is highly consistent. Bar charts indicating the proportional richness and abundance of taxa in 55 soil samples sequenced with both 454 and Illumina MiSeq at the phylum, class, and order levels. (TIF) [file pone.0090234.s004.tif]
